# ‘I can't go to her when I have a problem’: sexuality communication between South African adolescent girls and young women and their mothers

**DOI:** 10.1080/17290376.2022.2060295

**Published:** 2022-04-20

**Authors:** Zoe Duby, Wilmé Verwoerd, Katja Isaksen, Kim Jonas, Kealeboga Maruping, Janan Dietrich, Ashleigh Lovette, Caroline Kuo, Catherine Mathews

**Affiliations:** a Health Systems Research Unit, South African Medical Research Council, Cape Town, South Africa; b Division of Social and Behavioural Sciences in the School of Public Health and Family Medicine, University of Cape Town, Cape Town, South Africa; c Centre for International Health, University of Bergen, Bergen, Norway; d Perinatal HIV Research Unit, University of the Witwatersrand, Johannesburg, South Africa; e Department of Behavioral and Social Sciences, Brown University School of Public Health, Providence, RI, USA

**Keywords:** Adolescent girls and young women, South Africa, sexuality communication, parents

## Abstract

Parent–adolescent sexuality communication, the process in which parents and their adolescent children discuss sexuality and sexual and reproductive health, is a key component for adolescents’ protective behaviours. Open communication with parents, particularly mothers, enables informed sexual and reproductive health (SRH) decision-making amongst adolescent girls and young women (AGYW). As part of a qualitative study evaluating a South African combination HIV prevention intervention for AGYW, we explored perspectives on SRH communication among AGYW and mothers of AGYW, and the effects of the intervention on sexuality communication as perceived by AGYW, mothers of AGYW, intervention facilitators and implementers, and community leaders. In-depth interviews and focus group discussions were conducted with 185 AGYW aged 15–24 years who had participated in the intervention, seven mothers of AGYW intervention recipients, 14 intervention facilitators, six community leaders, and 12 intervention implementers. Key themes that emerged in analysis were (1) Barriers to Sexuality communication, (2) Implications of Gaps in Sexuality Communication, and (3) Addressing Barriers to Sexuality communication. Barriers to sexuality communication included inability or unwillingness to discuss sex, a generation gap, proscriptive socio-cultural guidelines, and mothers’ discomfort, lack of knowledge and self-efficacy, and fear of encouraging promiscuity. AGYW described making poorly-informed SRH decisions alone, expressing a desire for more open communication with and support from parents/mothers. Framed within the social cognitive theory, these findings can help to guide efforts to address barriers around parent–adolescent sexuality communication, inform interventions aimed at targeting SRH issues amongst AGYW, such as unintended pregnancy and HIV, and support meaningful engagement of parents in supporting AGYW in navigating pathways to achieving their SRH goals.

## Introduction

South Africa has the largest HIV epidemic in the world, with an estimated 7.8 million people living with HIV (STATS SA, 2020). A quarter of all new infections occur amongst adolescent girls and young women (AGYW) aged 15–24 years, at a rate more than double that of young men (UNAIDS, 2019). The disproportionate HIV risk faced by AGYW can be attributed to a number of structural and environmental factors including gender inequality, gender-based violence, gender discrimination, gender norms, and taboos about sexuality, which combine to negatively impact the ability of AGYW to protect themselves from HIV and other sexually transmitted infections (STIs), prevent unintended pregnancy, seek health services, and make informed decisions about their sexual and reproductive health (SRH) and lives (UNAIDS, 2019). Additionally, many AGYW lack knowledge of and access to SRH services and commodities, which combined with social and economic factors, also contribute to high rates of early and unintended pregnancies (Jonas et al., 2020; STATS SA, 2020).

Early pregnancy is associated with risks to the health and well-being of adolescent girls (Panday, Makiwane, Ranchod, & Letsoalo, 2009). As with rates of HIV, South Africa also has high rates of teenage pregnancy; in 2016, 16% of females between 15 and 19 years had begun childbearing (National Department of Health, Statistics South Africa, South African Medical Research Council, ICF, 2019). Evidence suggests that AGYW lack comprehensive and accurate knowledge about the sexual transmission of HIV (Simbayi et al., 2019; UNAIDS, 2019).

### Parent–adolescent sexuality communication

Studies have demonstrated strong associations between parent/caregiver-child communication around sex and adolescents’ protective attitudes/behaviours, including condom and contraceptive use, fewer sexual partners, delayed sexual debut, and the ability to discuss sexual risk with partners (Biddlecom, Awusabo- Asare, & Bankole, 2009; Crosby, Hanson, & Rager, 2009; O’Donnell & Fuxman, 2017; Panday et al., 2009; Soon et al., 2013). ‘Safe-sex behavioural competence’, the ability to negotiate and enact protective behaviours, amongst AGYW has been associated with frequent communication with their mothers (Mastro & Zimmer-Gembeck, 2015). To enable AGYW to make safe, informed decisions about relationships, sex and contraceptive use, they require accurate information and support (Mastro & Zimmer-Gembeck, 2015). Given the high rates of teenage pregnancy, HIV, STIs, and low uptake of contraception in South Africa, parent–adolescent communication, specifically mother-AGYW communication, may be particularly important in this context (Kuo et al., 2016).

There has been increased focus on the importance of parent-based adolescent sexual health interventions, and their potential for improving SRH (Akers, Holland, & Bost, 2011). However, there remain gaps in understanding the specific characteristics of these interventions, and their effects on sexuality communication, particularly in the South African context (Santa Maria, Markham, Bluethmann, & Mullen, 2015). Additionally, very few of the studies on parent/caregiver-adolescent sexuality communication around sex have specifically examined the mother-daughter dyad, particularly in the sub-Saharan African context. Exploring this dyadic relationship in terms of sexuality communication in the South African context is critical for understanding the role that mothers can play in addressing SRH challenges among AGYW.

### Theoretical and conceptual framework

There has been a call for increased consideration and use of social and behavioural theories to understand barriers to sexuality communication, explore mediating pathways and moderators, and therefore design more effective adolescent SRH interventions (Santa Maria et al., 2015). Firstly, ‘communication’ can be understood to be a dynamic and contextual interpersonal process, requiring certain behavioural skills to be successful in achieving its intention of transmitting a message from a sender to a receiver in order to bring about a desired response (McCabe & Timmins, 2013). ‘Parent–adolescent sexuality communication’ can be framed as an ongoing, iterative, interpersonal process in which parents impart information and values about sex and relationships to their adolescent children, and discuss aspects related to sexuality, inclusive of sexual feelings and behaviour, contraceptives, and sexual and reproductive health (Graham Holmes, Strassberg, & Himle, 2020). This process can be unidirectional or can involve dialogue. Parent–adolescent sexuality communication occurs at different levels and takes different forms, for example, the provision of factual and biological information about the reproductive system, conversations about contraception and pregnancy, and broader sexuality issues such as homosexuality and sexual consent (Fitzharris & Werner-Wilson, 2004).

The social cognitive theory, based social cognitive constructs, namely self-efficacy and outcome expectancies, is useful as a framework with which to understand parent–adolescent sexuality communication (DiIorio et al., 2000). Mothers’ resistance to discuss sex with adolescents is most often based on their own lack of knowledge/skills, lack of self-efficacy and self-perceived skills for effective communication, socio-cultural norms around sexuality communication, and fear that communication will encourage sexual behaviour (Davis, Blitstein, Evans, & Kamyab, 2010; Guilamo-Ramos, Jaccard, Dittus, & Collins, 2008; Jaccard, Dodge, & Dittus, 2002; Seif, Kohi, & Moshiro, 2018). Within this framework, it makes sense that parents who feel confident, or possess self-efficacy and the belief that they are able to discuss SRH issues with their adolescents, and believe that the discussions will lead to positive outcomes, are more likely to communicate effectively (DiIorio et al., 2000).

### The intervention

Protecting AGYW from the risks of early pregnancy, STIs, and HIV requires a holistic approach, which addresses the social, economic, and structural factors preventing them from practicing safe and consensual sex. South Africa's National Strategic Plan (NSP) for HIV, TB, and STIs sought to address a number of these issues by prioritising the provision of comprehensive targeted combination prevention interventions. In addition to access to HIV treatment and services, the NSP specified that targeted interventions should include the provision of appropriate social support which specifically includes information and emotional support from people including parents. The idea being that with such support, AGYW have a greater likelihood of receiving the information and assistance they need for safe SRH choices.

The Global Fund invested in a South African combination HIV prevention intervention for AGYW in ten South African districts purposively selected to include some of the most vulnerable AGYW in the country, with the highest HIV incidence (The Global Fund, 2018). The intervention was implemented 2016–2019, and comprised an intensive, comprehensive package of components aimed at reducing new HIV infections and rates of teen pregnancy amongst AGYW through the provision of HIV, TB, and SRH services, counselling, and education. The HERStory study was an evaluation of the intervention, which aimed to assess how the intervention and its impacts were perceived by AGYW and their communities, describe the factors that determined these perceptions, and describe the successes and failures of the intervention. In addition the qualitative component of the evaluation aimed to describe the factors that influence AGYW decision-making around sexual and reproductive health, and the effects of intervention participation on these. The findings presented in this paper derive from the qualitative evaluation, specifically of the intervention components that provided AGYW in and out of school, with SRH education, peer support, behaviour change counselling, life skills, empowerment activities, and aspects designed to build self-esteem and self-confidence. Further information about the intervention and the overall evaluation can be found at: https://www.samrc.ac.za/intramural-research-units/HealthSystems-HERStory.

In examining the perspectives of AGYW who had participated in the intervention, mothers of AGYW intervention recipients, community leaders, as well as intervention facilitators and implementers, this paper presents valuable narratives of sexuality communication between AGYW and their mothers from multiple settings in South Africa. Describing intervention participants’ and other stakeholders’ perceptions of how participating in an intervention affected this communication, these findings can help to inform future interventions, and contribute to addressing barriers in sexuality communication.

## Methods

Data presented in this paper were collected between August 2018 and March 2019 in five South African districts: City of Cape Town, Western Cape (WC); Uthungulu, KwaZulu-Natal (KZN); Gert Sibande, Mpumalanga (MPU); Bojanala, North West (NW); and Nelson Mandela Bay, Eastern Cape (EC). Respondents were purposively selected to take part in individual in-depth interviews (IDIs) of 20–40 minutes, and focus group discussions (FGDs) of 40–90 minutes each with 4–8 participants. A total of 185 AGYW aged 15–24 who had participated in the intervention took part in 57 IDIs and 19 FGDs. In addition, IDIs were conducted with seven mothers of AGYW intervention recipients, 14 intervention facilitators, six community leaders, and 12 intervention implementers (Table 1).

**Table 1.** Qualitative sample per site for the HERStory qualitative study component.

Province	Western Cape (WC)	KwaZulu-Natal (KZN)	Mpumalanga (MPU)	North West (NW)	Eastern Cape (EC)	
District	City of Cape Town	King Cetshwayo	Gert Sibande	Bojanala	Nelson Mandela Bay	
Characteristic	Urban	Rural	Semi-urban	Semi-urban	Urban	
**Sample Group**	***N***	***N***	***N***	***N***	***N***	**Total *N***
AGYW* Intervention participants 15–19 years	47	13	33	16	38	147
AGYW Intervention participants 20–24 years	11	20	0	7	0	38
Mothers	1	2	0	1	3	7
Facilitators	0	5	4	2	3	14
Community Leaders	0	2	1	2	1	6
Implementers	2	2	3	3	2	12

*AGYW = Adolescent Girls and Young Women.

A team of trained and experienced female researchers conducted IDIs and FGDs in participants’ language of choice (English, isiZulu, isiXhosa, seTswana, or siSwati) using semi-structured topic guides. Interview guides included open-ended questions and probes for potential additional issues, allowing for iteration, probing, and digression on relevant themes. Discussion relating to parent–adolescent sexuality communication arose in response to questions around sources of advice and support for AGYW for relationship and health concerns. Specific questions in the AGYW topic guide included: Who do you talk to about relationship problems?; Who do you talk to about health concerns? Parents were asked questions including To what extent do you discuss health and well-being / HIV / pregnancy / condoms with your daughter?; What kinds of things do you say to your daughter about health and well-being / HIV / pregnancy / condoms?; How easy or difficult is to discuss health and well-being / HIV / pregnancy / condoms with your daughter?; What kinds of things make it easy / difficult? No specific question on sexuality communication were included in the topic guides for intervention facilitators, implementers, or community leaders.

Informed consent was obtained from all participants 18 years and older. All participants 18 years and older were informed of the purpose and process of research before being asked to provide their informed, written consent. For those younger than 18 years, written assent with written guardian consent was obtained. Reimbursement and refreshments were provided. The study protocol and research tools were approved by the South African Medical Research Council Research Ethics Committee, and by the Associate Director for Science in the Center for Global Health in the Centers for Disease Control and Prevention.

### Data analysis

Audio recordings of IDIs and FGDs were transcribed verbatim into their original language, reviewed by the interviewer/s for accuracy, translated into English and re-reviewed. Thematic analysis of transcribed interviews followed an integrated and cyclical approach. After the initial codebook development based on study objectives and interview guides, codes underwent inductive refinement as the initial analysis phase progressed (Bradley, Curry, & Devers, 2007; Nowell, Norris, White, & Moules, 2017; Vaismoradi, Jones, Turunen, & Snelgrove, 2016). The analysis process included identifying and refining themes, coding with NVivo 12 software, and collaborative interpretation of the data through team members sharing insights from individual analysis and reflection during regular team discussions. The research team's interpretation of the data was verified and confirmed through member-checking feedback workshops held with AGYW intervention recipients (not necessarily those who had participated in IDIs and FGDs). During the workshops, the research team summarised and presented key themes and findings to the participants, who were then invited to give feedback, discuss their interpretation of the findings, and expand or elaborate on themes. The research team facilitated discussions on each theme, which were captured through notes and audio recordings, transcribed, reviewed and included in analysis.

## Findings

The findings presented below are arranged into key thematic areas that emerged during analysis and illustrate the perspectives on SRH communication among AGYW and mothers of AGYW, and the effects of the intervention on sexuality communication as perceived by AGYW, mothers of AGYW, intervention facilitators and implementers, and community leaders. The findings are arranged into three main thematic areas that emerged: (1) Barriers to Sexuality communication, (2) Implications of Gaps in Sexuality Communication, and (3) Addressing Barriers to Sexuality communication. Quotations are excerpts from English transcripts or translations; in brackets are details of the respondents’ site and sample group.

### Barriers to sexuality communication

#### Inability and unwillingness to discuss sex

Articulated in the narratives of adolescent girls and young women was the feeling of being unable to communicate with or seek emotional support or advice from their mothers about sexual, reproductive, and/or romantic issues. Attempts at conversations around sexuality were often met with negative and proscriptive discourse:

I cannot discuss sex issues with my mum because she’ll ask me … where did I even get the permission to sleep with a boy?. (KZN, AGYW 15–18 years)

Mothers’ discomfort in talking about sex led them to actively avoid the topic or speak in vague and ambiguous terms:

In these things (matters to do with relationships and sex), she is the one who speaks first … I would pretend as if I don't hear her. (EC, mother)

In the 15–18 years age group, AGYW reported avoiding speaking to their mothers about problems related to sex out of concern that assumptions that would be made about their sexual activity, thereby leading to disagreements and conflict. Furthermore, AGYW explained that if they admitted being involved in a romantic relationship or simply asked questions around sex, it would be met with anger and even violence. As a result, AGYW generally avoided discussing and/or seeking advice from their mothers due to fear of potential verbal and physical confrontation:

I don't speak to my mum because (she) will end up shouting at me saying that … now I’m into this love business. (KZN, AGYW 15–18 years)

Fear of accessing contraceptives due to negative reactions from mothers was also cited as a barrier by adolescent girls in the 15–18 age group:

It's not that girls don't want to prevent (use contraceptives), we do want, but … your mum will ask “are you sleeping with boys now?” … that makes you scared of going to prevent. (WC, AGYW 15–18 years)

In addition to communication barriers around sexuality generally, discussion pertaining to contraceptives and condoms was highlighted as particularly problematic:

I have never spoken to my mother about these issues … if I talk about going to prevent (use contraceptives), she would think I’m planning to have sex … if I want to check for HIV, she’d think I’ve had sex … she ends up hitting me and yelling at me. (KZN, AGYW 15–18 years)

#### Mothers’ fear of encouraging promiscuity

The perception that mothers avoided discussing SRH out of fear that doing so would encourage daughters’ sexual activity was articulated:

My mother does not want to talk to us about these things (SRH) because she feels like if she does, it is like she says we must do them (have sex). (EC, AGYW 15–18 years)

Mothers themselves expressed concern that having conversations about contraceptives might be perceived as an acceptance of their daughters’ sexual behaviour, encouraging promiscuity, and risk-taking behaviour:

If I talk to her about pregnancy things, it's like I’m sending her to the streets (encouraging promiscuity) … Even if she comes with this information, it feels as if I am sending her … I am afraid of sending her to do it. (EC, mother)

Speaking about contraceptives was interpreted as condoning sex:

You are giving the child a key (permission) … a chance to open her legs … giving her to the boys. (KZN, mother)

Mothers also seemed to specifically avoid any mention of condoms and contraceptives, fearing that it will condone sex and thus encourage promiscuous behaviour:

I don't want to get started with that (talking about condoms) because that will encourage her to do things (have sex) … like prevention (contraceptives) and so on. She will hear where she hears it (SRH information) but I don't want it to come out of my mouth … you don't have to give the child a key to go for prevention … because that gives a lot of ideas, she is seeing that ‘oh mother is allowing me to do that (have sex) … she's given me permission to do it because she told me to go and prevent’. (KZN, mother)

Mothers expressed the sentiment that using contraceptives would ruin their daughters’ innocence, and encourage them to have multiple partners:

If you say she can go for prevention … (you will) spoil the child … She will no longer date just one person because she knows that she can't get pregnant … She’ll have 1, then 2, then 3 (boyfriends) [laughing] … keep on counting, she knows that she will not get pregnant. (KZN, mother)

Mothers felt that they did not want to play a part in enabling their daughters’ sexual activity:

I won't allow my daughter to do family planning … ! (by letting her use contraceptives) … you have given her the armour … it will be easy for her to sleep around. (MPU, mother)

### Socio-cultural guidelines around sexuality communication

#### Generation gap

A common theme in discussions with AGYW respondents pertained to their impression of parents as ‘old fashioned’ and from ‘a different era’:

It's really difficult, our parents still live in the past. (WC, AGYW 15–18 years)

AGYW believed that parental avoidance of conversations around sex and contraception was due to them being old and outdated:

Most parents are old … they are afraid to talk to their children … and tell them (about sex). (KZN, AGYW 19–24 years)

Lack of parental support for AGYW use of contraceptives was described as being a result of their outdated SRH knowledge and old-fashioned views:

The attitude that the parents have … (asking) ‘How can you be on the injection?’, they don't think about what might happen in the future … mistakes can happen, and using contraceptives might be helpful if unplanned situations occur … during their times … most of them got pregnant at a young age, they were not provided with the information that we get today. (MPU, AGYW 15–18 years)

The generational knowledge gap was also acknowledged by mothers, who felt that they lacked sufficient knowledge themselves:

Our children bring flyers (with SRH information) home from school … we are not educated enough. (MPU, mother)

Lack of knowledge and/or courage on the parents’ part contributed to the barriers in communication:

We are scared to talk to our children … scared because we don't know where to start … they are still young. (KZN, mother)

In addition to the generational gap in how topics around sex are approached/perceived, proscriptive socio-cultural guidelines around sexuality communication also inhibit open communication:

It is still very difficult (for mothers) to talk to their children … Some mothers can see that their child has started sleeping out, but it they find it difficult to confront them

(MPU, Community Leader). In some communities, overt discussion of sexual matters is regarded as unacceptable, especially in front of adolescents and young people:

Once in our community, the people from health (department) were invited (to provide SRH information) … But you know they only talk a little bit, because we as Africans we get angry when people talk about things such as (sex) … especially older people, they say we should put certain topics aside and not talk about them … in public … and the new generation also attend these meetings … So when old people attend they get upset saying ‘No, why must we talk about things such as sex and AIDS in front of the children?’ (NW, mother)

### Implications of gaps in sexuality communication

Implications of the lack of communication between mothers and daughters about sexual issues include AGYW's lack of access to accurate and sufficient information required to make informed SRH decisions. Lacking advice and information, many AGYW end up making their own poorly-informed decisions:

I cannot speak to my mother … I’m scared of her … I prefer not to speak to her and just make my own decisions. (KZN, AGYW 15–18 years)

The lack of support and information increases AGYW vulnerability:

Some (girls) are unable to get information (about sex), that's why at the end their lives they are messed up. (KZN, AGYW 19–24 years)

Uninformed decision making can result in unplanned pregnancy and increased HIV risk:

If you don't have a person to talk to, you can end up being pregnant at an early age … you can contract diseases because of lack of knowledge. (MPU, AGYW 15–18 years)

The sentiment that high rates of teenage pregnancy are attributable to a lack of sexuality communication and support from parents was also expressed by a mother:

The problem with parents today … kids feel they can't speak to them about anything, so they try and explore different avenues on their own … that's where the problem comes in … communication is a big problem … there are a lot of kids … whose parents don't speak to them about anything at all … so we have a lot of teenage pregnancy in the area. (WC, mother)

One respondent articulated her experience of poor timing in sexuality communication, describing how her mother only started talking to her about contraceptives after she’d already got pregnant during her first sexual encounter:

I did not have a lot of information because … my mum and I … (only) started … to be open … after (my) pregnancy … She told me that I should prevent and you must do this and that … after pregnancy, so I felt that if they could have maybe told me about these things when I was still growing up maybe at the age of 14, 15, I would not have fallen pregnant … Since the person who made me pregnant was my ‘virgin breaker’ (first sexual partner) … I don't think I had enough information. (NW, AGYW 19–24 years)

Some adolescent girls in the 15–18 age group also felt that parents’ restrictive attitudes towards romantic and sexual issues, sparked an even greater curiosity and incentive to engage in forbidden activities:

When we start going out with boys and our parents keep reprimanding us about this, we tend to do the very opposite of what we are told not to do. We are curious to find out what is it that our parents don't want us to do. (WC, AGYW 15–18 years)

The inability to speak candidly to parents, combined with a fear of recrimination, led some AGYW to hide from parents and take greater risks:

We would like to tell our parents when we are going to see our boyfriends but we can't … when we actually decide to go out, we wonder who to inform … We end up not coming back home for fear of being punished. (WC, AGYW 15–18 years)

Receiving information and support around SRH, contraceptive use, and relationships from parents was perceived by AGYW respondents to be unusual.

### Addressing barriers to sexuality communication

Despite the significant barriers the AGYW faced in communicating with their parents, they reported a need for and desire to communicate more openly with their parents about romantic and sexual issues and suggested that parents need to be engaged and supported in order to address these barriers, and enable effective communication:

Our parents need awareness … they just keep quiet and they don't teach us … (interventions need) to bring our parents together for awareness so that (they) … talk to us. (EC, AGYW 15–18 years)

It was suggested that health facilities could assist in educating parents:

(At the clinic), when educating about contraceptives … they should also educate our parents to be calm towards their kids, so that they feel free to discuss any problems they are facing, talk with them about sex matters, and maybe advise on what to use so that you don't fall pregnant. (MPU, AGYW 19–24 years)

In particular, AGYW expressed a need for parents to have more open, non-aggressive responses to discussions about sexual issues with their adolescents:

Parents need to be taught … not to beat their children but to talk to them … because when you decide to tell your parent (about contraceptive use), they will think that you are sexually active and you are sleeping around. (MPU, AGYW 19–24 years)

There was an acknowledgement that mothers have valuable personal experiences that they could share with their daughters, but at the same time concerns that their knowledge was largely outdated or inaccurate. AGYW felt that their mothers would benefit from being provided with accurate and up-to-date information, in order to address the generation gap and improve parental understanding of the realities of their daughters’ lives, and the need to support contraceptive use. Based on their own experiences of participating in the intervention, adolescent girls in the 15–18 age group suggested that parents would also benefit from being engaged in SRH education interventions, to improve understanding of AGYW SRH issues, update their knowledge with regards to contraception and health services, and in turn help to close the generation/knowledge gap, making it easier for AGYW to seek their support:

Our parents should participate in the same programme that teaches us … about pregnancy and stuff, so that when the time comes for us to talk about these issues they can understand. (WC, AGYW 15–18 years)

Parental support and open communication was described as critical for AGYW healthy decision making:

Parents must talk to their children, because if parents don't speak to their kids who will? Some make wrong decisions because they not advised by their parents … (my mother) said no (she won't tell me about sex) because she will be encouraging it, I told her that if I wanted to do it I would … it is your duty as a parent to advise me. (EC, AGYW 15–18 years)

Other community stakeholders agreed on the importance of engaging and educating parents in communication around SRH issues:

We have to advise, especially mothers, to change from their old ideas … A mother must be able to speak about whatever is happening in the life of a girl child … (not) keep quiet … Then we will win that battle (HIV and teenage pregnancy). (MPU, Community Leader)

One of the intervention facilitators spoke of the paucity of SRH knowledge amongst parents, and the necessity of equipping parents with this knowledge, so that they can also understand the SRH information their daughters receive:

This subject that we are covering (in the intervention) is very important … teenage pregnancy, STIs and sometimes parents don't know this stuff, so we need to equip parents by having meetings with them … so that they can also know what the girls are learning. (EC, Intervention Facilitator)

Since many parents, especially in rural areas have little formal education themselves, providing them with accurate and up to date SRH information is necessary if interventions for AGYW are to be successful:

The parents, they need to understand why the programme is there for their children. Remember, we also give them material … on teenage pregnancy, contraception … I am talking about rural areas, most or some of them (parents) have never been to school (so) when you give them that information and you share with them, they see the benefit and as much as they understand, they will also encourage their children, say ‘ … as a parent I will encourage you’ … we just need to continuously have that space … we cannot win this if the parents are not involved. (NW, Intervention Implementer)

#### Mothers’ recognition of importance of sexuality communication

One mother explained the importance of openly discussing issues of sex and contraception with her daughters, despite the difficulty in doing so:

You (should be) able to sit the child down and warn her that my child the danger of having sex at early is this … the after effects … sit down … share and discuss the issue of prevention … ‘it's fine, I can take you to get an injection … and also you must use a condom’ … unfortunately I had no parent to guide me … so I always advise my children. (NW, mother)

Despite mothers’ concerns about discussing SRH issues, in particular contraceptives, there were some who acknowledged the importance of overcoming discomfort in order to speak openly and honestly and provide guidance for their daughters’ SRH choices and behaviours:

Us as women (need) to take initiative to sit down with our children … be open … so when it happens that (my daughter) tells me ‘Mama now I have a feeling to have sex’ or ‘I slept with someone’ … I must sit down with her, even though it will hurt me … because I don't what sex activity really happened, did they use protection or what … ? … But I must cross question her, to say ‘ok … fine you had sex … explain as to whether you used a protection’ … she will have to tell me … so that I can guide her. (NW, mother)

### Effects of the intervention on sexuality communication between AGYW and their mothers

According to some respondents, SRH communication between AGYW and their mothers was improved as a result of the intervention, specifically components designed to provide SRH and rights education, and activities designed to empower AGYW, and increase their self-esteem and self-efficacy. Some AGYW respondents felt that the interventions had given them the courage to speak openly with parents about SRH issues. Possessing accurate SRH information themselves, AGYW felt better prepared and more empowered to talk to their mothers:

(Being part of the intervention) made it easier to share with my mother … When I first started my periods, I had already learnt in school about it so I know what was going on … it was easier to tell my mum about it … (from) there … me and my mother started talking about boyfriends and girl stuff. (WC, AGYW 15–18 years)

Participation in the intervention was also perceived to have improved communication on the part of AGYW:

I used to hide my things … I was not open to my mother … because I was afraid of how she would respond … (after attending intervention) I ended up opening to my mother. (EC, AGYW 15–18 years)

AGYW felt more empowered to raise SRH topics of conversation:

I learned how to deal with my mother because when I would talk to her about some things, she would keep quiet – I would tell her not to keep quiet, because if she is not going to advise me on these things then who will? (EC, AGYW 15–18 years)

Feeling better equipped to deal with parents’ adverse reactions to questions/discussions around sexuality, enabled AGYW to negotiate initial communication barriers and have open, informative discussions:

Now … I’m able to talk to my mum about everything … things that are going on in my life, maybe my partner and I are having issues, I can sit down with my mum and tell her … I’m able to say to her, ‘mama you’re older than me … what did you do?’ … we talk about condoms … we talk about everything … before (the intervention) I felt like if I start talking about condoms, my mum will say I’m sleeping around … I felt like … she will judge me … since … I’ve been in (the intervention) … I’m able to talk about everything with her … she gives me advice. (NW, AGYW 19–24 years)

In turn, open communication enabled healthier decision making:

‘I used to … not tell anyone about things, I kept everything to myself … now I found help because I can see that keeping things to yourself makes you do things you never thought you will do (take more risks). (EC, AGYW 15–18 years)

Amongst AGYW there was a sense that the nature of relationships with their parents had been positively impacted through their participation in the intervention, enabling them to share potentially stigmatising details of their lives:

(Being part of these interventions) has taught me to share, because I used to think that if I shared with my mother she will judge me, but now I share with her … It's much better because now we talk about everything, she even told me that when she grew up she also had the same experience as I do now. (WC, AGYW 15–18 years)

In addition, a newly acquired ability to disclose non-heterosexuality to parents was articulated by one respondent:

I am a lesbian. For some time I was afraid to come out of the closet, and even when they learnt about my sexuality on Facebook I continued to deny it. Even when people started talking about my sexuality in my street I continued to deny it … I did not know where to start or how should I inform my parents … (after attending this programme) … I sat down with them and told them of my sexuality and they understood. (WC, AGYW 15–18 years)

A new-found ability to discuss SRH matters more openly also led to some AGYW having greater access to and support for using contraceptives from their mothers:

My mum did not want me to prevent (use contraceptives), (but when) I joined (the programme) and explained to her, so (now) she lets me prevent. (WC, AGYW 15–18 years)

Participating in the intervention programme also connected AGYW to alternative channels of information, support and advice:

Since I joined (intervention) I was able to talk … cause I wasn't able to talk … about condoms and stuff, I did not know how to share that with anyone … . but (being part of intervention) has made us to be free (to discuss these things). (WC, AGYW 15–18 years)

The sentiment was expressed by one AGYW that participating in an intervention of this nature earlier, would have prevented her unintended pregnancy:

If I had been an (intervention) member before I fell pregnant, I wouldn't have a child, even now because … I would’ve been able to communicate with my mother. (NW, AGYW 19–24 years)

Parents of AGYW who had participated in the intervention also felt that there had been positive impacts:

Sometimes she hides some things … she is shy to talk about some things with me as her parent, but at (intervention programme) they speak openly without holding stuff back … now because she speaks openly, there is nothing hidden … now I can speak with her … (this intervention) has made it easy. (EC, mother)

The knowledge that their daughters were receiving SRH information facilitated more open and honest conversations, and thus enabling parents to better support their daughters:

It's not easy (speaking with my daughter about sex), but when she is attending such programmes it becomes easy, because then I am talking about something she knows … We need people like you (interventions) … maybe parents will learn a lot for their children, because some kids rush into these things, and have regrets later, and it becomes your responsibility … So we parents want these programmes also, because they help us with our kids. (EC, mother)

## Discussion

These findings indicate that despite the recognised need for communication about sexuality, substantial barriers to communication exist between South African AGYW and their mothers. Although respondents’ narratives suggested that many barriers to sexuality communication between AGYW and their mothers remain, our findings indicate that interventions can be successful in addressing some of these barriers, and that by reducing sexuality communication barriers, AGYW access to contraceptives may be improved. The social cognitive theory constructs of self-efficacy and outcome expectancies help to frame our findings relating to barriers to AGYW-mother sexuality communication, which in turn can help to inform the design of interventions. One additional aspect revealed in our analysis, which the social cognitive theory does not account for, relates to the socio-cultural context in which these interpersonal/dyadic communication interactions take place. Norms around sexuality communication emerged in our findings as a key barrier, highlighting the need to consider cultural context when designing interventions.

### Barriers to AGYW-parent sexuality communication

#### Mothers’ lack of self-efficacy

Mothers interviewed in our study described their reticence in discussing sexual and relationship matters with their daughters, partly due to a sense of being ill-informed themselves, lacking courage to do so, and out of concern that speaking about preventing pregnancy would be interpreted as an encouragement of their daughters’ sexual activity. Conversations around sexuality create feelings of discomfort and whereas adolescents may fear reprimand from parents, parents may also lack courage and/or information, and fear that talking about sex will be interpreted as encouragement (Duby et al., 2015; Isaksen & Sandøy, 2019; Mudhovozi & Ramarumo, 2012). Evidence suggests that lack of knowledge and skills, combined with generational and educational gaps between parents and adolescents, also contribute to parents’ sense of disempowerment, lack of self-efficacy, and reluctance to discuss SRH with adolescents (Panday et al., 2009).

Although parental SRH knowledge alone is insufficient to influence the communication behaviour, it is an important prerequisite (Seif et al., 2018). Aggressive or punitive reactions to adolescents’ attempts to discuss SRH with parents, as described by AGYW in our study, may be related to this sense of disempowerment and lack of self-efficacy (Panday et al., 2009). As respondents in our study explained, due to discomfort and a feeling of having inadequate information and skills, mothers deflect daughters’ attempts to discuss SRH. This concurs with evidence suggesting that self-efficacy is a significant predictor of communication around sexual behaviour in mother-daughter dyads (Davis et al., 2010).

#### Mothers’ outcome expectancies

Mothers in our study expressed the sentiment that it is unnecessary to discuss sexual issues with their daughters because they were too young to be sexually active, and sexuality discussions would promote earlier sexual debut and promiscuity. Literature from South Africa and other countries in the region confirms this widespread reluctance of parents to talk to their adolescent children about SRH; this reluctance is often explained in terms of a fear that communication will encourage early initiation of sexual activity and the belief that school-aged children are not having sex and thus there is no need (Agbemenu et al., 2018; Kuo et al., 2016; Mpondo, Ruiter, Schaafsma, van den Borne, & Reddy, 2018; Muhwezi, 2015; Okigbo, Kabiru, Mumah, Mojola, & Beguy, 2015). Prior research from South Africa has demonstrated that maternal figures generally fail to recognise the contribution of their own behaviours and motivations on AGYW risk behaviour, failing to consider the implications of their daughters’ fear of discussing sex and contraception with them (AVAC, 2018; Panday et al., 2009).

Additionally, mothers in our study expressed a disconnect with the reality of their daughter's sexual and romantic lives, as displayed in the generation gap referred to by mothers and daughters alike. It is likely that mothers’ unfamiliarity with the AGYW context means that while the intent to protect their adolescents is positive, the parenting role and associated norms may lead to ineffective outcomes (AVAC, 2018). The complexity of relationships between AGYW and their mothers was a re-occurring theme within which was embedded discussion of how AGYW sexual behaviour could be both a cause and consequence of relationship tensions. This aligns with existing literature suggesting that a lack of connectedness and poor quality relations between parents and adolescents is linked with sexual risk behaviour, including early sexual debut, amongst adolescents (Markham et al., 2010; Peltzer, Mngqundaniso, & Petros, 2006).

#### AGYW outcome expectancies

AGYW in our study described their fear of reprimand, and even physical punishment, if they were to raise the sexuality related topics with their parents/mothers. AGYW who expect that engaging parents in SRH discussions may result in punishment or lessons promoting abstinence are unlikely to disclose anything about their SRH to their mothers (AVAC, 2018; Mpondo et al., 2018; Wamoyi et al., 2010).

#### Generation gap

Both parents and AGYW in our study explained that discomfort stemming from a generational gap in views, and lack of understanding on the parents’ part, prevented open sexuality communication. Linked to AGYW outcome expectancies described above, when AGYW view their parents’ attitudes as inflexible, outdated and irrelevant, and expect that engaging parents in SRH discussions will be fruitless or reactionary, AGYW avoid such interactions. Interventions for AGYW SRH in South Africa need to be sensitive towards socio-cultural norms, the traditional morals, and expectations of the older generation, while emphasising the importance of communication across generational boundaries (Nilsson, Edin, Kinsman, Kahn, & Norris, 2020).

#### Socio-cultural norms

Socio-culturally informed behavioural norms and linguistic guidelines around sexuality communication in much of sub-Saharan Africa frame sex as a taboo topic, to be discussed using euphemistic terms, or only in socially sanctioned situations (Cain, Schensul, & Mlobeli, 2011; Duby et al., 2015; Kawai et al., 2008; Mpondo et al., 2018; Okigbo et al., 2015; Wight et al., 2006). Socio-cultural norms restricting sexuality communication impede open discussion of SRH matters between adolescents and young people and their parents (Bastien, Kajula, & Muhwezi, 2011; Kuo et al., 2016; Seif et al., 2018). Adolescent sexuality is often viewed as something that needs to be controlled and repressed, and thus sexuality communication between parents and adolescents in the sub-Saharan African region tends to be punitive, focusing on abstinence, rather than information about contraceptives and/or condoms (Mudhovozi & Ramarumo, 2012; Panday et al., 2009; Rodgers, Tarimo, McGuire, & Diversi, 2018).

### Implications of barriers in AGYW-parent sexuality communication

Evident in the narratives of AGYW was a sense of emotional isolation, and the consequential negative impact on their ability to make safe decisions regarding their SRH, including contraceptive use and HIV testing. As described by AGYW respondents in our study, the fear of reprimand or punishment prevents them from seeking support from or confiding in their mothers/caregivers, so instead seek advice from peers, who are equally misinformed, or choose to make SRH decisions on their own. Those AGYW respondents in our study who had already been pregnant, suggested that had they received better information and been able to communicate with their parents about sex earlier on, they would have been more likely to avoid early pregnancy. Where communication between parents and adolescents is limited, adolescents tend to turn to their peers; however peer support and advice often lacks confidentiality and accurate information (AVAC, 2018; Vilanculos & Nduna, 2017). AGYW perceptions of the lack of trust and empathy that their mothers have in their decision making, drives them away from these maternal figures, who have the potential to be positive behaviour influencers, towards negative ones, also resulting in the formation of inaccurate views and high-risk practices related to sexual health (AVAC, 2018).

### Need for improved AGYW-parent sexuality communication

AGYW in this study voiced a desire for improved communication with, and increased emotional support from, mother/parents/caregivers regarding SRH. Adolescents often chose parents as their preferred source of SRH information and desire more opportunities to discuss SRH issues in a safe and positive manner (Awusabo- Asare, Bankole, & Kumi-Kyereme, 2008; Mabunda & Madiba, 2017; Namisi et al., 2013; Soon et al., 2013). Despite their reported discomfort, parents in this study also recognised the need to communicate more openly about sexual issues with their adolescents. The role of parents in the SRH choices and behaviours of adolescents is increasingly being recognised. Parents, as key players in the sexual socialisation of adolescents and young people, are in a position to communicate with their children about sex, having direct access and an ability to frame discussions in accordance with their social and developmental context (Jaccard et al., 2002; Muhwezi, 2015; Poulsen et al., 2010; Widman, Choukas-Bradley, Noar, Nesi, & Garrett, 2016).

### Effects of the intervention

Framing our findings within the social cognitive framework, the effects of intervention participation on AGYW were related to (1) an improved sense of empowerment and self-efficacy to initiate sexuality communication, ask for support from their mothers, and speak openly about their SRH concerns; and (2) AGYW being empowered to prepare themselves for negative outcomes including parents’ adverse reactions, to questions/discussions around sex.

These findings suggest that through the provision of sexual and reproductive health and rights education, and activities designed to empower AGYW, interventions can be successful in addressing some of the barriers to open sexuality communication.

### Recommendations

Based on our findings, and previous research on parent–child sexuality communication, by addressing the barriers to open communication, future interventions can incorporate more meaningful engagement of parents in adolescent SRH. Parent-delivered SRH information, education and support may be an efficacious strategy to prevent unintended pregnancies and the HIV infection amongst AGYW (Crosby et al., 2009). However, as seen in this study, there are many factors that need to be considered; the parent–adolescent relationship, the way in which parents communicate SRH messages, the timing of discussions, the type and accuracy of the information they provide and the ways in which the information is perceived and interpreted by AGYW. Considering the high HIV prevalence and teenage pregnancy rates in South Africa, all possible avenues for reducing AGYW sexual risk must be explored. Interventions addressing the barriers to parent–child SRH communication may be a feasible approach to reducing adolescent sexual risk, but would require support and training for parents (Namisi et al., 2013).

#### Improve parents’ communication skills

Adolescents are more likely to talk with parents about sex and see them as valid resources for information about sex when they perceive parents as ‘open’ and responsive (Grossman, Sarwar, Richer, & Erkut, 2017). By assisting parents to engage in positive parenting styles which encourage open, attentive, and responsive communication about sex, interventions targeting adolescent SRH may be augmented (Isaksen & Sandøy, 2019). Importantly, parents should be taught communication skills beyond simply imparting accurate information to their adolescents; evidence suggests that skills such speaking less, listening more, asking questions, and providing non-judgmental support enables parents to overcome adolescents’ resistance to and discomfort in communicating about SRH with their parents, promoting a sense of trust (Akers et al., 2011; DiIorio et al., 2000).

#### Improve parents’ self-efficacy by enhancing knowledge and skills

In order to address barriers relating to parents’ self-efficacy, it would be beneficial for interventions to include strategies that provide parents/caregivers with a knowledge base and skill-set, to enhance their motivation and confidence in communicating around SRH topics with their adolescents, and create opportunities for communication and collaboration among parents/caregivers (AVAC, 2018; Guilamo-Ramos et al., 2008; Seif et al., 2018). The provision of knowledge to parents is one key ingredient, but must be combined with efforts to empower parent/caregivers, validating and addressing their potential discomfort towards talking about sex, and working to build their courage in discussing SRH, which will in turn increase their ability to be responsive to the needs of adolescents and support them to make safe and informed decisions about sex (Grossman et al., 2017; Nilsson et al., 2020).

#### Bridge the empathy gap

In order to reinforce positive behaviours amongst AGYW, there is a need for empathetic support networks that provide holistic advice and support for managing relationships and SRH overall, rather than focusing only on HIV prevention (AVAC, 2018). For this to be successful, there is a need to ‘bridge the empathy gap’ by helping parents/maternal figures to understand the AGYW context and the implications of their attitudes and communication style on AGYW decision making (AVAC, 2018). Maternal figures are more likely to be able to effectively communicate with AGYW if they have more realistic expectations regarding AGYW relationship and sexual behaviours, framing their role as supporting AGYW to navigate relationships, in order to overcome their own concerns about promoting sex (AVAC, 2018).

#### Contextually relevant interventions

Within South African cultural frameworks, maternal figures are expected to play the role of sexuality socialising agents of appropriate behaviours to girl-children, and are thus in a position to provide accurate SRH information to their daughters, and equip them with the knowledge and skills necessary for safe-sex competence, empowering them with the ability to make informed decisions regarding the engagement in protective behaviours (Mastro & Zimmer-Gembeck, 2015; Mudhovozi & Ramarumo, 2012). Interventions need to be designed and implemented in a manner that recognises the importance of communication across generational boundaries, is sensitive to generational norms, culturally and linguistically appropriate, and works to address socio-cultural norms and religious beliefs that may create resistance to open, inter-generational communication around sex (Nilsson et al., 2020). Further in-depth research to enrich understanding of how sexuality communication should best be included in SRH interventions in the South African context would be beneficial.

## Limitations

Limitations of this study include the small sample size of parents interviewed, the lack of representation of fathers/paternal figures, and the fact that we were not able to interview AGYW-mother dyads together. The research team faced challenges accessing parents of AGYW, to invite them to take part in the study. Firstly, interviewers relied on AGYW participants to be willing for their parent/mother/caregiver to be contacted; secondly, even when successfully contacted, very few parents were willing and/or available to take part in interviews, hence the small sample size. It should also be noted that although the term ‘Adolescent Girls and Young Women’ (AGYW) is employed in HIV prevention programming, it would be beneficial if adolescent girls and young women were disaggregated, as the SRH needs, developmental phases, social status, expectations and experiences of these age groups are different. Notably, the majority of literature relates to parent–adolescent communication and it does not include young adult women. The lack of clearer disaggregation in our analysis is noted as a weakness. Respondents in this study were overwhelmingly supportive of the need for and benefits of parent–child sexuality communication may have been due to intervention effects; at the time of data collection, several of the AGYW and mothers had been exposed to a number of the interventions (for up to two years). Furthermore, the overwhelmingly positive reports on the impacts of the intervention may have in part be due to social desirability bias. Although the research team was independent from the intervention, it is possible that participants viewed the interviewers as connected to the implementers, and therefore shared positive opinions. Additionally, data was collected at one time point, which means that the narratives of intervention recipients may be prone to recall bias.

## Conclusions

Our findings corroborate those from prior studies demonstrating that interventions can be successful in reducing barriers to sexuality communication, and that with support, mothers can and will discuss SRH with their adolescents (Panday et al., 2009). Systematic reviews of adolescent HIV prevention and sexual risk reduction interventions in South Africa identified a clear gap, with no empirically tested family interventions for HIV prevention and sexual risk reduction substantively involving parents (Kuo et al., 2016). Pilot interventions have since shown beneficial effects on parent–child communication and parent self-efficacy in condom use, as well as beneficial effects in parenting involvement and sex communication (Kuo et al., 2016). Our findings add to the evidence base suggesting that it is possible to overcome norms that prohibit open sexuality communication, and in turn provide a more enabling environment for AGYW to make informed decisions about sex (Svanemyr, Amin, Robles, & Greene, 2015).

There is a need to leverage AGYW ecosystems to support prevention and risk reduction by addressing communication barriers between AGYW and their parents/caregivers, facilitating more effective support to AGYW in their SRH decision-making. The findings from this study provide valuable insight into the dynamics of sexuality communication in the mother-daughter dyad in South Africa. Based on these findings and on evidence suggesting the important role that mothers can play in addressing SRH challenges among adolescent and young women, future interventions need to incorporate more meaningful engagement of mothers, and other caregivers, to facilitate an reduction in sexuality communication barriers and consequently enable more effective support for AGYW in their SRH decision-making. Open channels of communication, accurate sources of SRH information, and social support network required to enable AGYW to make safe and healthy SRH decisions and adopt prevention practices are critical but missing.

## Data Availability

The data that support the findings of this study are available on request from the corresponding author, ZD.
